# Acute Intermittent Porphyria Labeled Initially As Guillain-Barre Syndrome: Challenging Diagnosis

**DOI:** 10.7759/cureus.48753

**Published:** 2023-11-13

**Authors:** Hassan A Alayafi, Talal K Aljumah, Omar B Alluhayyan, Ali Alqaraishi, Sarah A Aljwair

**Affiliations:** 1 Neurology, King Abdulaziz Medical City, Ministry of National Guard Health Affairs, Research Center, King Abdullah International Medical Research Centre, Riyadh, SAU; 2 Internal Medicine, King Abdulaziz Medical City, Ministry of National Guard Health Affairs, Research Center, King Abdullah International Medical Research Centre, Riyadh, SAU

**Keywords:** pres, neuropathic porphyria, ivf, gbs, acute intermittent porphyria

## Abstract

Acute intermittent porphyria (AIP) is an autosomal, dominant, rare metabolic disturbance that results from a defect in the activity of the heme biosynthesis. It has a heterogeneous presentation, making a prompt diagnosis challenging. We report a case of acute intermittent porphyria in a young female who underwent in vitro fertilization (IVF) and presented with recurrent abdominal pain and posterior reversible encephalopathy syndrome (PRES), progressing to acute progressive quadriparesis post-delivery.

## Introduction

Porphyrias are an inherited group of disorders manifested by the precipitation of intermediate metabolites due to enzymatic defects in the heme biosynthesis [1]. Acute intermittent porphyria (AIP) is a rare disorder with a prevalence of around five cases per 10000 individuals [2]. Females are more likely to develop symptoms, especially during periods with alterations in the level of sex hormones such as ovulation, menstruation, and pregnancy [3]. Depending on which enzyme is deficient, the presentation, severity, and prognosis differ. AIP is the most severe form. Typically, patients present with recurrent, poorly localizable abdominal pain. Other associated manifestations include autonomic dysfunction and neurological symptoms, most commonly peripheral neuropathies [4]. Posterior reversible encephalopathy syndrome (PRES) is an acute or subacute cerebral syndrome. Patients usually present with visual obscurations, seizures, and varying degrees of encephalopathy [5]. It is usually associated with hypertension, infection/sepsis, autoimmune disease, or pre-eclampsia/eclampsia. It is a clinical and radiological diagnosis. The presence of patchy, contrast-enhancing T1-weighted images and high signal intensity on T2-weighted and fluid-attenuated inversion recovery (FLAIR) without diffusion restriction due to vasogenic edema is suggestive of PRES. Multiple case reports have highlighted patients presenting with porphyria and PRES [6,7]. However, the underlying association remains unknown, making a prompt diagnosis difficult. We here report a case of acute intermittent porphyria in a young female who underwent IVF induction presenting with recurrent abdominal pain, PRES, progressing to acute progressive quadriparesis post-delivery.

## Case presentation

**Figure 1:**
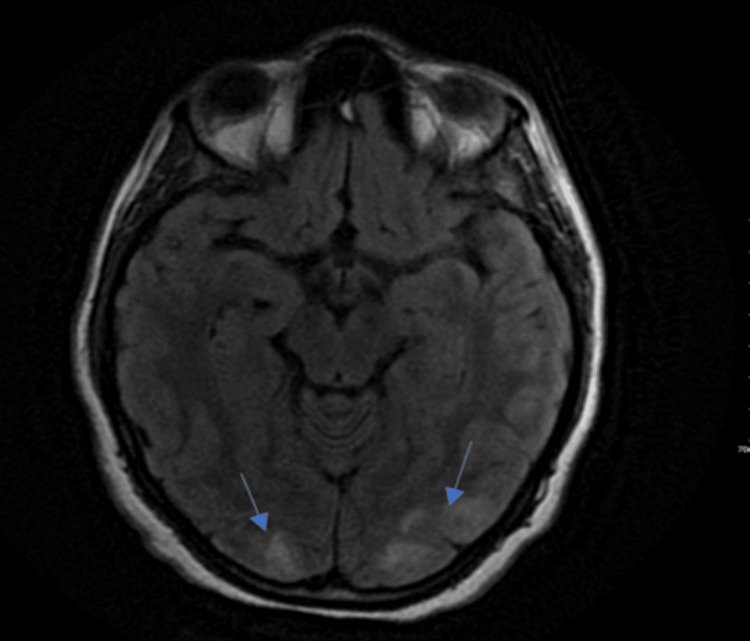
FLAIR MRI showing posterior high signal intensity changes FLAIR: fluid-attenuated inversion recovery

A 33-year-old female with primary infertility and polycystic ovarian syndrome for 10 years, underwent three trials of IVF induction over 10 years preceding this pregnancy until she finally got pregnant with triplets. At 23 weeks of gestation, she developed severe lower abdominal pain. She was admitted and treated as a case of urinary tract infection. At the 24th week of gestation, she went into premature labor, delivered her babies, and went home in stable condition. Within the next week, her abdominal pain worsened, so she returned to the hospital. She underwent dilatation and curettage for suspected retention of products of conception and then developed a fever, followed a few days later by visual disturbance and an episode of generalized tonic-clonic seizure. She was treated as a case of meningitis and was started on empirical anti-meningeal coverage and phenytoin for her seizures. After one week, she was transferred to our tertiary care center for further workup and a second opinion. Upon arrival, the patient's general and neurological examinations were unremarkable. Lumbar puncture (LP) was done and was negative for infection, so empirical anti-meningeal coverage was discontinued. Her brain MRI was consistent with PRES (Figure 1). She was treated supportively.

Over the next few days, the patient still complained of lower abdominal pain with lower limb pain. She also complained of visual and auditory hallucinations with severe insomnia. She was believed to have delirium vs. postpartum psychosis and was seen by mental health and started on olanzapine. However, the patient was not improving. Her labs revealed hyponatremia of 126 (mEq/L). The hyponatremia was questioned to be related to olanzapine or due to syndrome of inappropriate antidiuretic hormone (SIADH) as a complication of PRES. During that time, the patient was able to ambulate, and her level of consciousness was preserved. However, her hyponatremia was worsening despite fluid restriction and sodium chloride tablets. Therefore, she was admitted to the intensive care unit (ICU) for hypertonic saline via a central line. On the third day of her admission to the ICU, her sodium normalized, and she was downgraded to a regular ward. During that time, the patient started complaining of generalized pain, fatigue, and weakness. Her examination showed a motor power of 4 with intact reflexes. The patient was placed on forced vital capacity and negative inspiratory pressure. The measurements continued to be suboptimal as the patient was not giving full effort, even though her respiratory rate, oxygen saturation, and venous blood gas were always within normal.

She was admitted to the ICU again as her hyponatremia returned, and she became severely confused. The next day, the patient developed acute onset progressive quadriparesis, areflexia, facial diplegia, and weak neck flexors, she was not able to move her head on her own, and she had poor respiratory effort, as she was not able to achieve average forced vital capacity or a negative inspiratory pressure; however, she was able to maintain her oxygen saturation without CO_2_ retention. The ICU decided against intubation, as it might affect her future prognosis as she was maintaining. However, one day later, in the morning, the patient went into critical condition and developed tachycardia and tachypnea, requiring 15L via a nonrebreather mask; she was intubated by the ICU team. Since her clinical status was not stable, LB was not secured. Based on the clinical picture, she was treated empirically with plasma exchange as a case of Guillain-Barré syndrome (GBS). Intravenous immunoglobulin (IVIG) was contraindicated, as she had right upper limb thrombosis provoked by central line insertion in her previous ICU stay. Given the patient’s history of lower abdominal pain, acute quadriparesis with areflexia, hyponatremia, and psychiatric manifestations, the diagnosis of porphyria was considered. Therefore, serum and urinary porphobilinogen were sent out.

**Figure 2:**
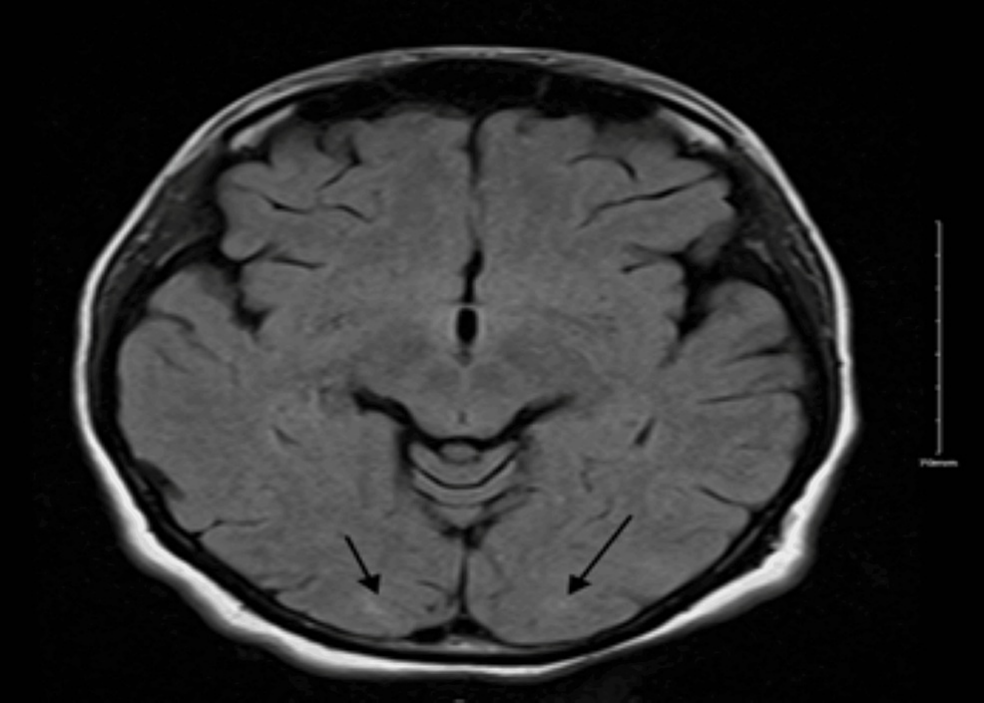
FLAIR MRI done one week after the initial showing a resolution of the high signal intensity changes FLAIR: fluid-attenuated inversion recovery

A nerve conduction study (NCS) and electromyography (EMG) were done three days after her flaccid paralysis, and they were completely normal. Cervical and lumbar spine MRI at the time was unremarkable. Brain MRI showed a resolution of PRES (Figure 2).

Three weeks after the onset of her symptoms, her NCS was repeated, which showed findings of severe sensorimotor polyneuropathy consistent with the acute motor sensory axonal neuropathy (AMSAN) variant. EMG was not done, as the patient was on therapeutic enoxaparin (Tables 1, 2). Porphyria workup came back three weeks later and revealed high porphyrins in erythrocytes (112 μg/dl; reference range < 60 μg/dl) with a urinary porphobilinogen (PBG) level of 805 μmol/24h (reference value up to 8.40 μmol/24h). The hematology team was consulted and the diagnosis was confirmed. IV hematin treatment was started, and the patient received a total of eight doses as per hematology recommendations. The patient is currently showing minimal improvement from a neurology point of view. She requires ventilatory support and is planning for an extensive rehabilitation program.

**Table 1:** Nerve conduction study for sensory responses NR: no response

Type of study	Nerve	Recording site	Latency (ms)	Amplitude (mV)	Conduction velocity (ms)
Sensory	Left Median	Wrist-Digit II	NR	NR	NR
Sensory	Right Median	Wrist-Digit II	NR	NR	NR
Sensory	Left Ulnar	Wrist-Digit V	NR	NR	NR
Sensory	Right Ulnar	Wrist-Digit V	NR	NR	NR
Sensory	Left Radial	Forearm-Wrist	NR	NR	NR
Sensory	Right Radial	Forearm-Wrist	NR	NR	NR
Sensory	Left Sural	Calf-Lat Mall	NR	NR	NR
Sensory	Right Sural	Calf-Lat Mall	NR	NR	NR
Sensory	Left Superficial Fibular	14cm-Ankle	NR	NR	NR
Sensory	Right Superficial Fibular	14cm-Ankle	NR	NR	NR

**Table 2:** Nerve conduction study for motor responses ABP: abductor pollicis brevis, ADM: abductor digit minimi, EDB: extensor digitorum brevis, AHB: abductor hallicus NR: no response

Type of study	Nerve	Stimulation site	Recording site	Latency (ms)	Amplitude (mV)	Conduction velocity (ms)	F wave (ms)
Motor	Left Median	APB	Wrist	NR	NR		-
			Elbow	NR	NR	NR	
Motor	Right Median	APB	Wrist	NR	NR		-
			Elbow	NR	NR	NR	
Motor	Left Ulnar	ADM	Wrist	NR	NR		-
			Bel. Elbow	NR	NR	NR	
			Abo. Elbow	NR	NR	NR	
Motor	Right Ulnar	ADM	Wrist	NR	NR		-
			Bel. Elbow	NR	NR	NR	
			Abo. Elbow	NR	NR	NR	
Motor	Left Fibular	EDB	Ankle	NR	NR		-
			Bel. Fib Head	NR	NR	NR	
			Pop Fossa	NR	NR	NR	
Motor	Left Fibular	Tib. Anterior	Fib Head	NR	NR		-
			Pop Fossa	NR	NR	NR	
Motor	Right Fibular	EDB	Ankle	-	-		-
			Bel. Fib Head	NR	NR	NR	
			Pop Fossa	NR	NR	NR	
Motor	Right Fibular	Tib. Anterior	Fib Head	NR	NR		-
			Pop Fossa	NR	NR	NR	
Motor	Left Tibial	AHB	Ankle	NR	NR		-
			Knee	NR	NR	NR	
Motor	Right Tibial	AHB	Ankle	-	-	-	-
			Knee	NR	NR	NR	

## Discussion

Acute porphyrias are rare metabolic conditions arising from an inherited enzyme defect [8]. Acute porphyric attacks can be triggered by different exogenous factors such as fasting, certain medications, or hormonal fluctuations [9]. A few cases reported an association between AIP and IVF [2,10]. A typical attack starts with intense abdominal pain, and autonomic dysfunction, accompanied by psychiatric and neurological manifestations. Commonly, it presents with peripheral neuropathies but can present with central and autonomic nervous system dysfunction [11].

The characteristic pattern of porphyric neuropathy is primarily motor axonal neuropathy that appears in 20-68% of patients with porphyria [11]. The precise mechanism of acute neuropathic porphyria is poorly understood. However, it is related to porphyrin and porphyrin precursor accumulation [12].

Porphyric neuropathy usually presents as a sensorimotor axonal neuropathy with autonomic dysfunction. The muscle weakness is usually proximal and involves the upper limbs, with NCS/EMG showing motor > sensory involvement. Both entities can have autonomic dysfunction, making a diagnosis challenging. However, GBS patients have a symmetrical distal weakness with associated CSF evidence of cytoalbumin dissociation [12].

Electrolyte abnormalities, such as hyponatremia attributed to SIADH, are observed in up to 90% of patients with an acute porphyria attack [11]. The hyponatremia is hypothesized to be attributed to SIADH or damage to the supraoptic nucleus of the hypothalamus [13]. Diagnosis during an acute attack is based on laboratory findings, such as raised urinary and serum porphobilinogen, in the setting of high clinical suspicion [12].

The management of acute porphyria is dependent on the prevention of precipitating causes and early recognition to prevent morbidity and mortality. In severe attacks, hemin is the treatment of choice [9]. In milder and earlier cases, supportive therapy and IV dextrose are preferred treatments owing to lesser side effects [11].

## Conclusions

Neuropathic porphyria is an easily missed entity due to its lack of typical clinical presentation. Therefore, misdiagnoses or late diagnoses are common and ultimately delay the management and lead to morbidity and mortality. In retrospect, acute porphyria attacks should be taken into consideration in patients with persistent unexplained refractory abdominal pain and peripheral neuropathies.
